# Two pear phytocytokines PbePep4 and PdrPep6 interfamilially elicit broad-spectrum immunity against various pathogens in crops

**DOI:** 10.1093/hr/uhag027

**Published:** 2026-01-29

**Authors:** Hai-Ting Wei, Ke Feng, Fan Xu, Xin-Zhong Cai

**Affiliations:** Zhejiang Key Laboratory of Biology and Ecological Regulation of Crop Pathogens and Insects, Institute of Biotechnology, College of Agriculture and Biotechnology, Zhejiang University, 866 Yu Hang Tang Road, Hangzhou 310058, China; Hainan Institute, Zhejiang University, Sanya, Hainan 572025, China; Zhejiang Key Laboratory of Biology and Ecological Regulation of Crop Pathogens and Insects, Institute of Biotechnology, College of Agriculture and Biotechnology, Zhejiang University, 866 Yu Hang Tang Road, Hangzhou 310058, China; Hainan Institute, Zhejiang University, Sanya, Hainan 572025, China; Zhejiang Key Laboratory of Biology and Ecological Regulation of Crop Pathogens and Insects, Institute of Biotechnology, College of Agriculture and Biotechnology, Zhejiang University, 866 Yu Hang Tang Road, Hangzhou 310058, China; Zhejiang Key Laboratory of Biology and Ecological Regulation of Crop Pathogens and Insects, Institute of Biotechnology, College of Agriculture and Biotechnology, Zhejiang University, 866 Yu Hang Tang Road, Hangzhou 310058, China; Hainan Institute, Zhejiang University, Sanya, Hainan 572025, China

## Abstract

Plant elicitor peptides (Peps) are a class of endogenous phytocytokine that enhances plant innate immunity against diverse pathogens. They are widely distributed in the plant kingdom, yet their interfamily compatibility of Peps perception remains controversial. In this study, two pear (*Pyrus* L.) Peps, PbePep4 (*Pyrus betulifolia*) and PdrPep6 (*Pyrus ussuriensis × communis Zhongai*), were identified and their function in eliciting interfamily immunity was dissected. We found that PbePep4 and PdrPep6 improved resistance of pear leaves to fire blight caused by *Erwinia amylovora*. Exogenous treatment with PbePep4 and PdrPep6 activated various immune responses in pear leaves, including burst of reactive oxygen species, deposition of callose, phosphorylation of mitogen-activated protein kinase, and up-regulation of defense genes. Intriguingly, these two pear peptides were able to interfamilially trigger immune responses of plants from Brassicaceae and Cucurbiaceae families. Application with PbePep4 and PdrPep6 enhanced the resistance of Brassicaceae species *Arabidopsis thaliana* and *Brassica napus* to *Sclerotinia sclerotiorum*, and that of Cucurbiaceae species *Citrullus lanatus* to *Botrytis cinerea*. We demonstrated that the key of these peptides to induce immunity in cross-family species is associated with the conservation of the conformed motif at the C-terminal of Pep peptides and their six active binding sites in PEPRs in cross-family species from the Rosaceae, Brassicaceae, and Cucurbiaceae. Taken together, our findings not only solved the debate whether plant Peps can only stimulate immunity within the family, but also clarified the exploitation potential of pear Peps as broad-spectrum immune inducers to control disease in crops of at least three families.

## Introduction

Fire blight, a prototypical necrotrophic plant disease caused by *Erwinia amylovora,* is a devastating disease, which severely damages *Pyrus* species and hinders the development of pear industry. To date, the main prevention and control methods for pear fire blight are agricultural control and chemical control. Agricultural control mainly focuses on the cultivation of disease-resistant varieties, but these varieties are prone to losing resistance and have a long breeding period. Chemical control (using pesticides) may cause many environmental and social problems such as increasing the risk of food safety (Philippe *et al.*, 2021), disrupting the balance of agricultural biodiversity and ecosystems and developing drug resistance in pathogens [1]. Therefore, exploitation of immune inducer derived from plants that is not harmful to crops is a beneficial attempt for the green prevention and control of pear fire blight.

Facing environmental stresses, plant cells share a rapid response strategy to enhance their chances of surviving injuries by detecting damage-associated molecular patterns (DAMPs) and phytocytokines that themselves produce. DAMPs and phytocytokines are recognized as a danger signal in the organism to activate defense pathways [2–5]. Plant elicitor peptides (Peps) are increasingly recognized as prototypical phytocytokines due to their intracellular origin and release and maturation upon injury, which, in turn, rapidly triggers defense responses [6–9]. In *Arabidopsis thaliana* (hereafter Arabidopsis), the endogenous peptides AtPep1-8 mature from the conserved C-terminal portions of their precursor proteins PROPEP1-8, respectively [10, 11]. Pathogen-induced physical damage causes a sustained increase in the influx of Ca^2+^ into the cytosol, resulting in the activation of Ca^2+^-dependent type II metacaspases (MCs), which then cleave PROPEPs and release Peps [12, 13]. The SUMOylation of PROPEP1 promotes its cleavage via MC4 [14]. The generated Peps are perceived by Pep receptor 1 (PEPR1) and PEPR2, mediating an association of PEPR1/2 with BRI1-associated kinase 1 (BAK1) and Botrytis-induced kinase 1 (BIK1) to initiate a series of immune responses [15–19]. These responses include a burst of reactive oxygen species (ROS), the production of defense-related hormones jasmonic acid and salicylic acid, and the expression of immune response genes [20, 21]. Distinct PROPEPs exhibit both overlapping and unique expression patterns, and their transcripts are upregulated by a variety of signaling molecules in response to stimuli [10, 11]. For instance, cell wall damage induces the expression of PROPEP1 and PROPEP3 in Arabidopsis to control stress responses [22]. Furthermore, a variety of stressors have the potential to increase the concentration of cytosolic Ca^2+^, which, in turn, promotes the proteolytic maturation of PROPEPs by activating the enzyme activity of MCs [12, 13, 23]. Most of above findings were based on model plant Arabidopsis. Although the sequences of PROPEP and PEPR have been identified in the Rosaceae family, such as pear, apricot, apple, peach species, and PmPep1, MdPep3, and PbPep4 have been characterized in their role in plant immunity [6], the role of Peps in Rosaceae plant resistance to fire blight remains unknown.

After extensive studies during last three decades, it is widely accepted that Peps are conserved signals across diverse families to regulate plant defenses [6, 9]. For instance, Pep peptides from Cucurbitaceae, Brassicaceae, and Solanaceae families are capable to stimulate the ethylene production of plants within the same family [24]. Similarly, Pep peptides from different species in same family of Solanaceae or Leguminosae can mutually induce the production of terpene volatiles [25]. Combination of transiently expressing Arabidopsis AtPEPR1 from the Brassicaceae family and corn ZmPEPR1 from the Cucurbitaceae family and treatments of AtPep1 and ZmPep1, respectively, could catalyze the production of ethylene in *Nicotiana benthamian,* indicating that the downstream signaling mechanism of the kinase domain of PEPR is highly conserved [24].

Yet, a few of studies [24, 25], on the cross-family Pep peptides from limited number of plant families showed the interfamily incompatibility of Peps perception. For instance, Pep peptides from Solanaceae, Leguminosae, Crocidaceae, Cruciferae, and Solanaceae were not recognized by species outside of the plant families of origin ([24, 25]. These studies used only ethylene and volatile production to characterize the perception of Peps, while no other typical immune responses and final resistance to pathogens were examined. Whether Peps harbor cross-family immune elicitation function needs to be further clarified in more families of plants and their Pep peptides.

In this study, we elucidated the role of pear peptides PbePep4 (*Pyrus betulifolia*) and PdrPep6 (*Pyrus ussuriensis **×** communis Zhongai*) in inducing immunity against pear fire blight and revealed that these two pear peptides could stimulate immunity across Brassicaceae and Cucurbitaceae species, which highlights the function of plant peptide in stimulating immunity in cross-family species [9]. This study provides new insights into plant phytocytokine-triggered immunity and clarifies the exploitation potential of pear peptides PbePep4 and PdrPep6 as broad-spectrum immune inducers, consequently providing new strategies for crop disease management.

## Results

### Identification of pear families of Peps and PEPRs

#### PROPEP

**Figure 1:**
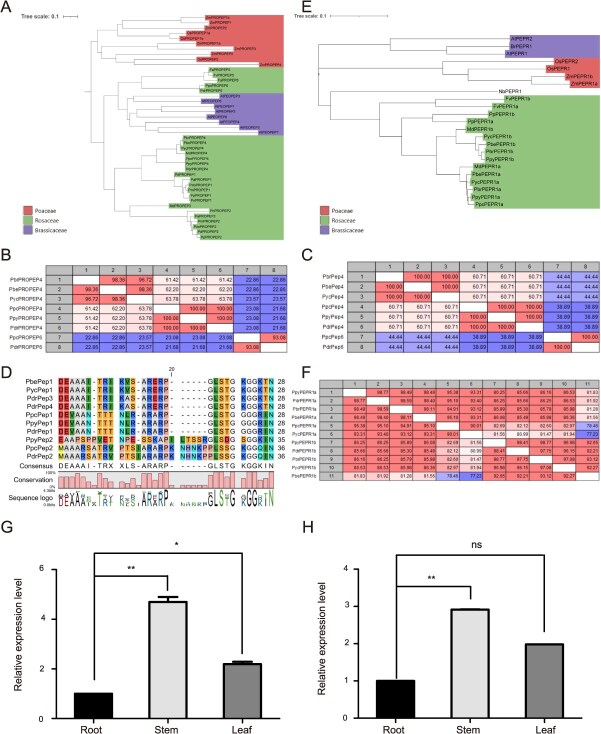
Identification of pear PROPEPS, mature PEPs, and PEPRs. (A) The phylogenetic tree of PROPEP proteins from Rosaceae, Brassicaceae, and Poaceae constructed by MEGA 12 software. (B) Homologous comparison of 7 PROPEP proteins in five pear varieties by CLC Main workbench software. (C) Homologous comparison of seven Peps in five pear varieties by CLC Main workbench software. (D) Conserved motif analysis of seven Peps. (E) The phylogenetic tree of PEPR proteins from Rosaceae, Brassicaceae and Poaceae constructed by MEGA 12 software. (F) Alignment of 7 PEPR proteins in five pear varieties by CLC Main workbench software. (G, H) Expression of *PbePEPR1a* (G) and *PbePROPEP4* (H) genes in root, stem, and leaf of *P. betulifolia*. The experiment was repeated three times. One-way ANOVA method was used to test the significance of differences among experimental groups. The data were shown as the mean ± SE. Star numbers represent levels of difference significance (^*^P < 0.05; ^**^P < 0.01. ns, not significant). Abbreviations of species in A–D: Pbe, *Pyrus betulifolia*; Ppc, *Pyrus pyrifolia Cuiguan*; Ppy, *Pyrus pyrifolia*; Pdr, *Pyrus ussuriensis **×** communis Zhongai*; Pyc, *Pyrus communis*.

Recently, PROPEP and PEPR sequences have been identified *in silico* in Rosaceae, increasing the number of known sequences to 18 PROPEP and 18 PEPR in 10 species [6]. Only one PROPEP (PbePROPEP4) has been identified in the genus *Pyrus* [6]. To more completely identify the Pep peptides of the genus *Pyrus*, bioinformatics analysis was performed on the PROPEP sequences of more species and varieties under the genus *Pyrus*. A BLASTp search was conducted using the *Arabidopsis* PROPEP protein sequence as a query against the Rosaceae genome database. Consequently, a total of seven *PROPEPs* were identified from pear varieties Duli, Cuiguan, Shali, Zhongdai No.1, and Xiyangli. A phylogenetic tree of PROPEP proteins from Rosaceae, Cruciferae, and Poaceae families were constructed, which showed that *PROPEPs* from the Rosaceae were clustered into two branches (highlighted in green in Fig. 1A). Among them, one branch containing PROPEP1/2/3/4 is unique, while the other carrying PROPEP5/6 was orthologs of those from Brassicaceae. The homology of PROPEP proteins from *Pyrus* plants was compared using CLC Main workbench software. The results showed that the homology was 98% for PbePROPEP4 and PycPROPEP4, 100% for PpcPROPEP4, PpyPROPEP4, and PdrPROPEP4, 93% for PdrPROPEP6 and PpcPROPEP6, while 20% for PROPEP4 and PROPEP6 (Fig. 1B). These indicated that there is a significant difference between PROPEP4 and PROPEP6 in *Pyrus*.

#### Mature PEPs

Seven mature peptide Peps were determined based on identified pear PROPEPs, which were classified into two classes: Pep4 and Pep6 (Table S1). The homology comparison analysis of the amino acid sequences of these seven pear Pep peptides was performed using the CLC Main workbench software. The amino acid sequences of PbrPep4, PbePep4, and PycPep4 were completely same, and so were PpcPep4, PpyPep4, and PdrPep4. The homology between these two types of peptides was ~60%. The homology between Pep4 and Pep6 is relatively low, around 40% (Fig. 1C). Conservation analysis was performed on these seven pear Pep peptides. A conserved motif among the 10 amino acids at the C-terminal of the Pep peptide was identified as LSxGxGGxxN (Fig. 1D).

#### Pear PEPRs

To date, previous studies have reported only one PEPR sequences (PbePEPR1a) in the genus *Pyrus* [6]. Here, bioinformatics analysis was also conducted on pears PEPRs. A BLASTp search using the *Arabidopsis* AtPEPR1 and AtPEPR2 as query against the genomic database of Rosaceae family resulted in identification of 9 PEPRs from five pear varieties, including Duli, Cuiguan, Shali, Zhongdai No.1, and Xueli. They were classified into two categories: PEPR1a and PEPR1b (Table S2). A phylogenetic tree of PEPR proteins from Rosaceae, Brassicaceae, and Poaceae families were constructed, showing that PEPRs from the Rosaceae gathered in one branch (Fig. 1E). The homology among five PEPR1as was above 90%, while the homology between PEPR1as and PEPR1bs was approximately from 77% to 86%. This indicated that compared to the PROPEPs, pear PRPRs among species of the genus *Pyrus* have relatively higher homology (Fig. 1F).

#### Expression patterns of *PbePEPR1a* and *PbePROPEP4*

To preliminarily examine whether the PROPEPs and PEPRs are functional, the tissue-specific expression patterns of *PbePEPR1a* and *PbePROPEP4* in the root, stem, and leaf of pear seedling were determined using real-time quantitative PCR (RT-qPCR). The results showed that *PbePROPEP4* and *PbePEPR1a* were expressed in the root, stem, and leaf. Of them, the expression of *PbePROPEP4* and *PbePEPR1a* in the stem were higher than those in the root and leaf (Fig. 1G and H).

### PbePep4 and PdrPep6 elicited immunity in pear

**Figure 2:**
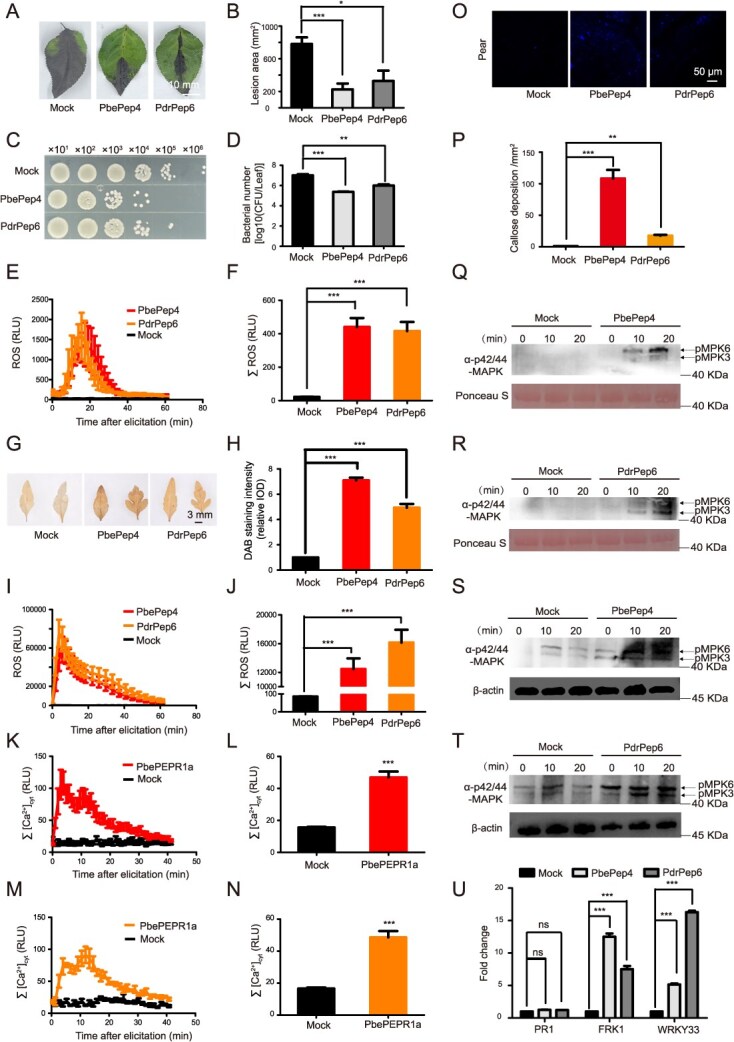
Pear peptides PbePep4 and PdrPep6 induced the resistance against pear fire blight. (A, B) Representative disease symptoms (A) and statistical analysis of lesion area (B) of *P. betulifolia* leaves were pretreated with PbePep4 and PdrPep6 at 10 days post inoculation with *E. amylovora*. (C, D) Measurement of bacterial count in the inoculated leaves (C) and statistical analysis (D). (E, F) ROS burst detected in *P. betulifolia* leaf discs treated PbePep4 and PdrPep6. The dynamic of ROS production (E) and statistics of ROS accumulation (F) were shown as mean values ± SE. (G, H) DAB staining of pear leaves treated PbePep4 and PdrPep6 for 24 hours (G) and the relative intensity of DAB staining (H) was measured using ImageJ. (I, J) ROS burst detected in tobacco leaf discs treated with PbePep4 and PdrPep6 after transient expression of PbePEPR1a on tobacco transformed with *Aequroin*. (K–N) Determination of Ca^2+^ influx induced by PbePep4 (K and L) and PdrPep6 (M and N) after transient expression of PbePEPR1a in tobacco transformed with Aequroin. (O, P) PbePep4 and PdrPep6 induce callose deposition in *P. betulifolia* leaves. Fluorescence microscopy imaging (O) and quantification (P) of the callose deposition were detected after pear leaves treated with PbePep4 and PdrPep6. (Q, R) PbePep4 (Q) and PdrPep6 (R) induced MAPK activation in tobacco after transient expression of PbePEPR1a. (S, T) PbePep4 (S) and PdrPep6 (T) induced MAPK activation in pear flowers. (U) Expression of *PR1*, *FRK1*, and *WRKY33* in *P. betulifolia* leaves upon treatment with PbePep4 and PdrPep6. One-way ANOVA method was used to test the significance of differences among experimental groups. At least three biological replicates were performed with similar results. For B, D, E, F, H, I, J, K, L, M, N, P, and U, the data were shown as the mean ± SE, Star numbers represent levels of difference significance (^*^, *P* < 0.05, ^**^, *P* < 0.01, ^***^, *P* < 0.001, ns, not significant).

Pep peptides are able to enhance the resistance of plants to pathogenic bacteria [6]. To further explore the role of pear peptides PbePep4 and PdrPep6 in disease resistance against fire blight, the leaves of pear were treated with 1 μM synthesized peptides PbePep4 and PdrPep6. The pathogenic bacteria of pear fire blight were needled and inoculated 24 hours after the treatment. The results showed that the disease severity of mock was significantly higher than those treated with two pear peptides (Fig. 2A and B). Consistent with this, the bacterial quantity of mock was significantly higher than that of PbePep4 and PdrPep6 treatments (Fig. 2C and D). These results showed that pear peptides PbePep4 and PdrPep6 were able to enhance the resistance in pear leaf against fire blight.

Next, we examined whether PbePep4 and PdrPep6 peptides can stimulate the early immune responses in plants, such as ROS burst, Ca^2+^ spiking, callose deposition, mitogen-activated protein kinases (MAPKs) activation, and immune gene expression. To verify whether pear peptides PbePep4 and PdrPep6 stimulate ROS burst in *Pyrus* leaves, the leaf discs were treated with PbePep4 or PdrPep6. We found that PbePep4 and PdrPep6 could stimulate ROS burst in the leaves of *P. betulifolia*, reaching the peak 15 minutes after treatment, while the control remained at a low level without a peak (Fig. 2E). The total accumulation of ROS showed that within 40 minutes of treatment with PbePep4 and PdrPep6, the accumulation of ROS could reach 400 relative light units (RLU) and 420 RLU, respectively. The accumulation of ROS in PbePep4 and PdrPep6 treatments was significantly higher than that in the mock (Fig. 2F). In parallel, the leaves of the pear soaked with PbePep4 or PdrPep6 were stained with diaminobenzidine (DAB). Leaves treated with pear peptides had brown spots (Fig. 2G). The H_2_O_2_ accumulation after PbePep4 and PdrPep6 treatments was significantly stronger than that in the mock (Fig. 2H). Together, these results indicated that pear peptides PbePep4 and PdrPep6 stimulate ROS burst in *Pyrus* leaves .

To explore whether pear peptides PbePep4 and PdrPep6 activate Ca^2+^ signaling, Ca^2+^ spiking was tested in Aequroin transgenic *N. benthamiana*. In this tobacco, PbePep4 or PdrPep6 at 500 nM alone could not stimulate neither the ROS burst nor the Ca^2+^ spiking (data not shown). However, after transiently expressing PbePEPR1a fused to GFP, the concentration of both ROS and Ca^2+^ increased rapidly after treatment with PbePep4 and PdrPep6 and was significantly higher than that in the mock (Fig. 2I–N). In conclusion, the transient expression PbePEPR1a in tobacco may recognize and bind to PbePep4 and PdrPep6, causing the Ca^2+^ spiking and ROS burst.

We also checked whether the two peptides can induce callose deposition, the leaves of *P. betulifolia* were soaked with PbePep4 and PdrPep6. This treatment induced large-scale deposition of callose in the leaves (Fig. 2O). The callose accumulation induced by both PbePep4 (100 mm^2^/unit area) and PdrPep6 (20 mm^2^/unit area) peptides was significantly higher than that of mock (<5 mm^2^/unit area; Fig. 2P).

To verify whether pear peptides PbePep4 and PdrPep6 activate MAPK phosphorylation, MAPK phosphorylation was determined after transient expression of PbePEPR1a in pear blossoms and *N. benthamiana* leaves. We found that MAPK6 could be strongly activated 20 minutes after treatment with PbePep4 and PdrPep6, while the activation of MAPK3 was relatively weaker *N. benthamiana* leaves when *PbePEPR1a* was co-expressed (Fig. 2Q and R). This suggested that PbePEPR1a recognizes and binds to pear peptides PbePep4 and PdrPep6, activating the phosphorylation reaction of MAPK. In pear blossoms, both PbePep4 (Fig. 2S) and PdrPep6 (Fig. 2T) could activate MAPK6 and MAPK3, and the level of activation was significantly stronger than that of mock. In summary, PbePep4 and PdrPep6 strongly induced MAPK phosphorylation.

For the purpose of determining whether pear peptides PbePep4 and PdrPep6 upregulate expression of defense genes, the leaves of pear were treated with the synthesized pear peptides and the expressions of pattern triggered immunity (PTI)-related genes, including the Systemic Acquired Resistance marker *PATHOGENESIS-RELATED GENE1*(*PR1)*, *FLG22-INDUCED RECEPTOR-LIKE KINASE1* (*FRK1)*, and *WRKY33*, were determined. Expression of *PR1* under PbePep4 and PdrPep6 treatments showed no significant difference from that in the mock (Fig. 2U), while the expressions of *FRK1* and *WRKY33* were significantly higher than that in the mock. PbePep4 treatment showed a higher upregulation for *FRK1* than PdrPep6 treatment, while PbePep4 treatment showed a lower upregulation for *WRKY33* than PdrPep6 treatment (Fig. 2U). These results indicated that pear peptides PbePep4 and PdrPep6 were able to induce the expression of defense genes in pear leaves.

To determine whether pear peptides PbePep4 and PdrPep6 inhibits the growth of *E. amylovora*, the bacterial culture was directly added with PbePep4 and PdrPep6. We found that the dynamic growth of *E. amylovory* treated with PbePep4 and PdrPep6 showed no significant difference compared to those in the mock, and the growth curves almost coincided (Fig. S1). The results indicated that neither pear peptides PbePep4 nor PdrPep6 inhibited the growth of pear fire blight pathogen, and thus confirmed that these peptides induced pear resistance to *E. amylovora* through activating diverse immune responses.

### PbePep4 and PdrPep6 interfamilially induced immune responses

#### PbePep4 and PdrPep6 interfamilially induced immune responses in cruciferous plants

**Figure 3:**
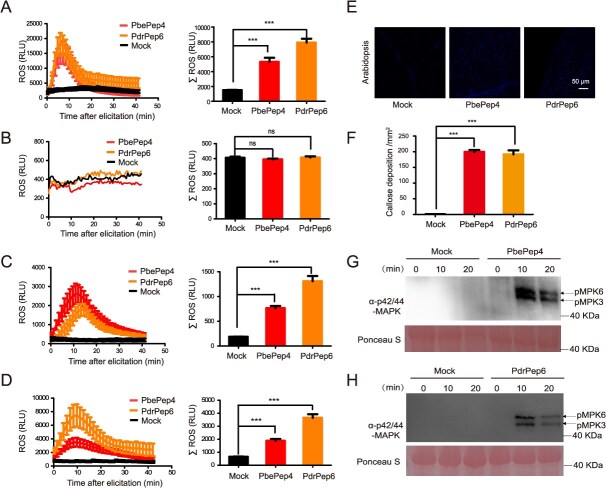
Pear peptides PbePep4 and PdrPep6 interfamilially induced the innate immunity in Brassicaceae species. (A) ROS burst detected in *A. thaliana* leaf discs treated with PbePep4 and PdrPep6. (B) ROS burst detected in *Atpepr1/2* mutant leaf discs treated with PbePep4 and PdrPep6. (C, D) ROS burst detected in *B. napus* (C) and *B. oleracea* (D) leaf discs respectively treated with PbePep4 and PdrPep6. (E, F) PbePep4 and PdrPep6 induce callose deposition in leaves of *A. thaliana*. Fluorescence microscopy imaging (E) and quantification (F) of the callose deposition were detected by aniline blue staining assay in leaves of *A. thaliana* and *C. lanatus* treated with PbePep4 and PdrPep6 for 24 hours. (G, H) MAPK activation was induced by PdrPep6 and PbePep4 in *A. thaliana*. MAP kinase phosphorylation was determined in *A. thaliana* leaves infiltrated with PbePep4 (G) and PdrPep6 (H). One-way ANOVA method was used to test the significance of differences among experimental groups. At least three biological replicates were performed with similar results. For A–D, and F, the data were shown as the mean ± SE. Star numbers represent levels of difference significance (^***^P < 0.001, ns, not significant).

We previously found that pear peptides PbePep4 and PdrPep6 activate ROS burst in *Arabidopsis* leaves. To verify whether they stimulate ROS burst in cruciferous plants, *Arabidopsis* wild-type and *Atpepr1/2* mutants, oilseed rape (*Brassica napus*), and cabbage (*Brassica oleracea*) were treated with synthesized PbePep4 and PdrPep6. ROS burst in *Arabidopsis* stimulated by PbePep4 and PdrPep6 reached the peak at about 7 minutes (left panel of Fig. 3A). Within 40 minutes after treatment, the total ROS accumulation in the PbePep4 treatment was 5,000 RLU, and PdrPep6 treatment 7,000 RLU. These levels of total ROS accumulation were significantly higher than that in the mock (right panel of Fig. 3A). As expected, PbePep4 and PdrPep6 did not trigger ROS burst in the *Atpepr1/2* mutant (Fig. 3B). These results indicated that both pear peptides PbePep4 and PdrPep6 interfamilially induce ROS burst in *Arabidopsis* in a PEPR-dependent manner.

PbePep4 and PdrPep6 also stimulated ROS burst in oilseed rape, reaching the peak point at about 13 minutes (left panel of Fig. 3C). Within 40 minutes after treatment, the total ROS accumulation upon both PbePep4 and PdrPep6 treatments was ~1,200 RLU, which were significantly higher than that in mock (right panel of Fig. 3C). Besides, the two pear peptides also stimulated ROS burst in cabbage, reaching the peak point at about 10 minutes (left panel of Fig. 3D). Within 40 minutes after treatment, the total ROS accumulation of PbePep4 was ~700 RLU, and PdrPep6 ~1,300 RLU, both of which were significantly higher than that of mock (right of Fig. 3D). In summary, these results indicated that both pear peptides PbePep4 and PdrPep6 interfamilially induce ROS burst in diverse cruciferous plants.

To verify whether pear peptides PbePep4 and PdrPep6 can interfamilially induce callose deposition of in cruciferous plants, *Arabidopsis* leaves were soaked with PbePep4 and PdrPep6. The treatments of PbePep4 and PdrPep6 could interfamilially induce callose deposition in *Arabidopsis* leaves (Fig. 3E and F).

For the purpose of testing whether PbePep4 and PdrPep6 can induce MAPK phosphorylation across families, *Arabidopsis* leaves were treated with PbePep4 and PdrPep6. We found that PbePep4 and PdrPep6 could interfamilially activate MPK6 and MPK3 in *Arabidopsis* (Fig. 3G and H).

#### PbePep4 and PdrPep6 interfamilially induced immune responses in Cucurbitaceae plants

**Figure 4:**
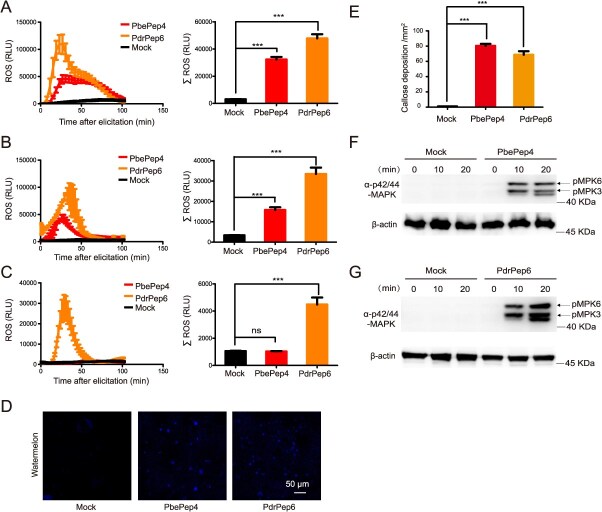
Pear peptides PbePep4 and PdrPep6 interfamilially induced the innate immunity in Cucurbitaceae species. (A–C) ROS burst detected in *C. lanatus* (A), *C. moschata* (B), and *Luffa aegyptiaca* (C) leaf discs respectively treated with PbePep4 and PdrPep6. The dynamic of ROS production (left panel) and statistics of ROS accumulation (right panel) were shown as mean values over 100 minutes ± SE. (D, E) PbePep4 and PdrPep6 induced callose deposition in leaves *C. lanatus*. Fluorescence microscopy imaging (D) and quantification (E) of the callose deposition were detected by aniline blue staining assay in leaves of *C. lanatus* treated with PbePep4 and PdrPep6 for 24 hours. (F, G) MAPK activation was induced by PdrPep6 and PbePep4 in *C. lanatus*. MAP kinase phosphorylation was determined in *C. lanatus* leaves infiltrated with PbePep4 (F) and PdrPep6 (G). One-way ANOVA method was used to test the significance of differences among experimental groups. At least three biological replicates were performed with similar results. For A, B, C, and E, the data were shown as the mean ± SE. Star numbers represented levels of difference significance (^***^P < 0.001, ns, not significant).

To verify whether pear peptides PbePep4 and PdrPep6 also interfamilially stimulate ROS burst in Cucurbitaceae, watermelon, pumpkin, and loofah leaves were treated with PbePep4 and PdrPep6. PbePep4 and PdrPep6 could stimulate ROS burst in watermelon (left panel of Fig. 4A). The peak point was reached 25 minutes after treatment, which took longer time than that in Arabidopsis (left panel of Fig. 4A) and the duration of ROS accumulation was longer (left panel of Fig. 4A). Within 100 minutes after treatment, the total ROS accumulation in PbePep4 and PbePep6 treatments were ~30, 000 RLU and 40, 000 RLU, respectively, both of which were significantly higher than that in the mock (right panel of Fig. 4A). Similarly, PbePep4 and PdrPep6 treatments stimulated ROS burst in pumpkins, with a peak point at ~25 minutes (left panel of Fig. 4B). Within 100 minutes after treatment, the total accumulation of ROS in the PbePep4 and PbePep6 treatments were ~14, 000 RLU and 30,000 RLU, respectively (right panel of Fig. 4B). Intriguingly, PdrPep6 stimulated ROS in loofah, reaching the peak point in ~25 minutes, while PbePep4 did not (left of Fig. 4C). Within 100 minutes after treatment, the total ROS accumulation in PbePep4 treatment was ~4,000 RLU, which was significantly higher than that in the mock (right panel of Fig. 4C). Taken together, these results indicated that both pear peptides PbePep4 and PdrPep6 interfamilially induce ROS burst in watermelon and pumpkin, but only PdrPep6 induces ROS burst in loofah.

The treatments of PbePep4 and PdrPep6 could also interfamilially induce callose deposition in watermelon leaves (Fig. 4D and E) and activate MPK6 and MPK3 in watermelon (Fig. 4F and G). PbePep4 activated a stronger MPK6 phosphorylation, and PdrPep6 activated the phosphorylation of both MPK6 and MPK3 (Fig. 4F and G).

### PbePep4 and PdrPep6 did not interfamilially induce ROS burst in Solanaceae or Poaceae plants

Next, we tested whether pear peptides PbePep4 and PdrPep6 stimulate ROS burst in Solanaceae and Poaceae, the leaves of *Solanum lycopersicum*, *Nicotiana tabacum*, *Capsicum annuum*, *Oryza sativa,* and *Zea mays* were treated with PbePep4 and PdrPep6. The ROS burst in tomato, tobacco, chili, rice, maize, mulberry trees, and willow trees stimulated by PbePep4 and PdrPep6 was comparable to that of mock, while ROS burst induced by flg22 was significantly higher than those induced by PbePep4 and PdrPep6 (Figs S2 and S3), indicating that PbePep4 and PdrPep6 did not interfamilially induce ROS burst in these Solanaceae or Poaceae plants.

#### AtPep1 and BnPep3 did not interfamilially induce ROS in plants of various families

To verify whether AtPep1 of *Arabidopsis* and BnPep3 of rapeseed can stimulate ROS burst in the Rosaceae, Cucurbitaceae, Solanaceae, and Poaceae plants, the leaves of *P. betulifolia*, *C. lanatus*, *C. moschata*, *L. aegyptiaca*, *S. lycopersicum*, *N. tabacum*, *C. annuum*, *O. sativa,* and *Z. mays* were treated with AtPep1 and BnPep3 at a final concentration of 500 nM to determine the ROS burst situation. ddH_2_O and flg22 were used as the negative and positive controls, respectively. The results showed that AtPep1 and BnPep3 showed no significant difference in the ROS stimulation of the leaves of those species, while flg22 was significantly higher than that of AtPep1, BnPep3, and ddH_2_O treatment, indicating that neither AtPep1 nor BnPep3 could interfamilially induce the burst of ROS in the Rosaceae, Cucurbitaceae, Solanaceae, and Poaceae plants (Fig. S4).

Collectively, our findings reveal that the ability to interfamilially induce immunity is restricted to Peps of a limited families including Rosaceae.

### PbePep4 and PdrPep6 interfamilially and thus broadly induced disease resistance in Cruciferous and Cucurbitaceae crops

**Figure 5:**
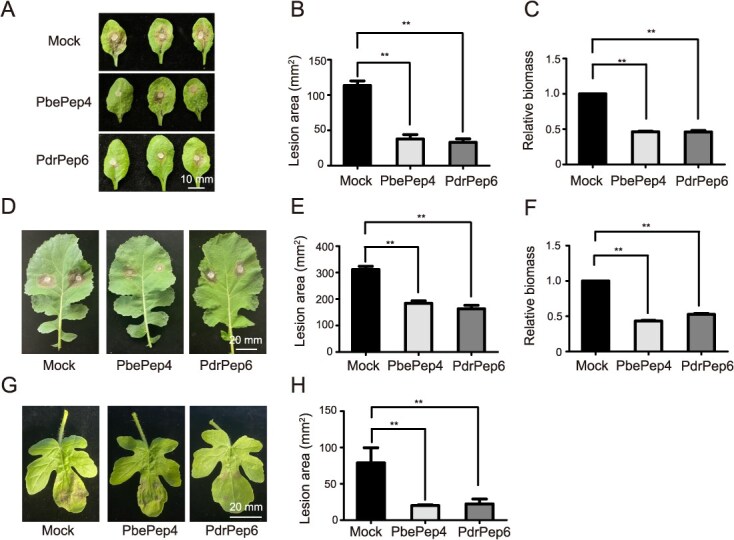
PbePep4 and PdrPep6 interfamilially induced the resistance against necrotrophic pathogens in Cucurbitaceae and Brassicaceae species**.** (A–C). Disease symptoms (A) and lesion area (B) of *A. thaliana* leaves pretreated with PbePep4 and PdrPep6 at 24 hours post-inoculation with *S. sclerotiorum*. *A. thaliana* leaves were pre-sprayed with PbePep4 and PdrPep6 at 24 hours before inoculation. Measurement of bacterial content in the inoculated leaves (C). (D–F). Disease symptoms (D) and lesion area (E) of *B. napus* leaves pretreated with PbePep4 and PdrPep6 at 24 hours post-inoculation with *S. sclerotiorum*. *B. napus* leaves were pre-sprayed with PbePep4 and PdrPep6 at 24 hours before inoculation. Measurement of bacterial content in the inoculated leaves (F). (G, H) Disease symptoms (G) and lesion area (H) of *C. lanatus* leaves pretreated with PbePep4 and PdrPep6 at 72 hours post-inoculation with *B. cinerea* spores. Leaves were pre-sprayed with PbePep4 and PdrPep6 at 24 hours before inoculation. One-way ANOVA method was used to test the significance of differences among experimental groups. At least three biological replicates were performed with similar results. For B, C, E, F, and H, the data were shown as the mean ± SE. Star numbers represented levels of difference significance (^**^P < 0.01, ^***^P < 0.001).

In order to investigate whether PbePep4 and PdrPep6 peptides interfamilially induce economically important disease resistance in plants, Sclerotinia stem rot in *Arabidopsis* and oilseed rape and gray mold in watermelon were tested upon treatments of PbePep4 or PdrPep6. The leaves of *Arabidopsis*, rapeseed, and watermelon were pre-treated with PbePep4 or PdrPep6. Sclerotinia plug and spore suspension of *B. cinerea* were inoculated on the leaves of *Arabidopsis* and oilseed rape and watermelon 24 hours after pre-treatment of peptides, respectively. The disease incidence and bacterial count of *Arabidopsis* and oilseed rape were measured 24 hours after inoculation, while watermelon leaves about 72 hours. We found that PbePep4 and PdrPep6 treatments obviously enhanced the resistance of *Arabidopsis* (Fig. 5A–C) and oilseed rape (Fig. 5D–F) against *S. sclerotinia*. In watermelons, the treatments of PbePep4 and PdrPep6 also enhanced the resistance of watermelons against gray mold (Fig. 5G and H). It is worthwhile to note that the pear peptides PbePep4 and PdrPep6 have no obvious inhibitory effect on the growth of *S. sclerotiorum* and *B. cinerea* (Fig. S5). In summary, PbePep4 and PdrPep6 peptides were able to interfamilially enhance disease resistance against economically-important necrotrophic pathogens in plants.

### Putative mechanisms of the interfamily immunity induced by PbePep4 and PdrPep6

**Figure 6:**
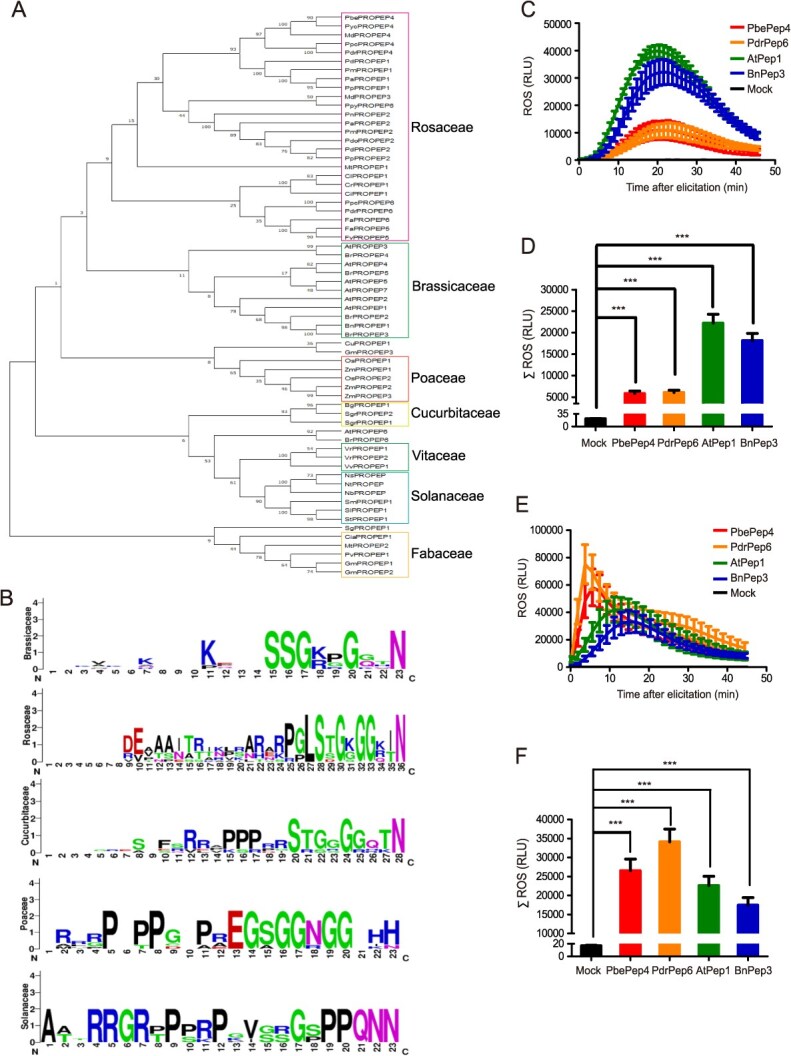
Pear Peps displayed high similarity in conformed motif with Peps from Brassicaceae and Cucurbitaceae plants. (A) The phylogenetic trees of PROPEP proteins in species of Rosaceae, Brassicaceae, Cucurbitaceae, Poaceae, Solanaceae, Fabaceae, and Vitaceae was constructed by MEGA 12. (B) Amino acid sequence alignment of peptides from species of Rosaceae, Brassicaceae, Cucurbitaceae, Poaceae, and Solanaceae. (C, D) ROS burst detected in tobacco leaf discs treated with PbePep4, PdrPep6, AtPep1, and BnPep3 after transient expression of *AtPEPR1*. The dynamic of ROS production (C) and statistics of ROS accumulation (D) were shown as mean values over 45 minutes ± SE. (E, F) ROS burst detected in tobacco leaf discs treated with PbePep4, PdrPep6, AtPep1, and BnPep3 after transient expression of PbePEPR1a. The dynamic of ROS production (E) and statistics of total ROS accumulation (F) were shown as mean values over 45 minutes ± SE. For C–F, the experiments were repeated at least three times. One-way ANOVA method was used to test the significance of differences among experimental groups. The data were shown as the mean ± SE. star numbers represent levels of difference significance (^***^, *P* < 0.001).

To understand the homology of PROPEPs from the plants in the Rosaceae, Cruciferae, Cucurbitaceae, Poaceae, and Solanaceae families, a phylogenetic tree of PROPEPs were constructed. The Rosaceae had the closest relationship with the Cruciferae, followed by the Poaceae, Cucurbitaceae, Vitaceae, Solanaceae, and Leguminous families (Fig. 6A). By analyzing amino acid sequences of Pep peptides from these five families, we found that the conformed motif at the C-terminal of Pep peptides from the Rosaceae, Brassicaceae, and Cucurbiaceae families was relatively similar, all having SxGxxGxxN. This might be one of the reasons why the PEP from Rosaceae had interfamily compatibility for the Brassicaceae and Cucurbiaceae (Fig. 6B).

Structurally, AtPEPR1 has 27 leucine-rich repeat (LRR) domain. The binding sites of AtPep1 and PEPR are in LRR4-LRR18 [26]. PbePep4, PdrPep6, AtPep1, and BnPep3 did not stimulate ROS burst in *N. benthamiana* (Figs S2 and S4). However, after transiently expressing AtPEPR1 or PbePEPR1a in *N. benthamiana*, PbePep4, PdrPep6, AtPep1, and BnPep3 all could stimulate ROS burst (Fig. 6C–F), suggesting that the binding sites of pear peptide PbePep4, PdrPep6 to PbePEPR1a may be the same with those of AtPep1 to AtPEPR1.

**Figure 7:**
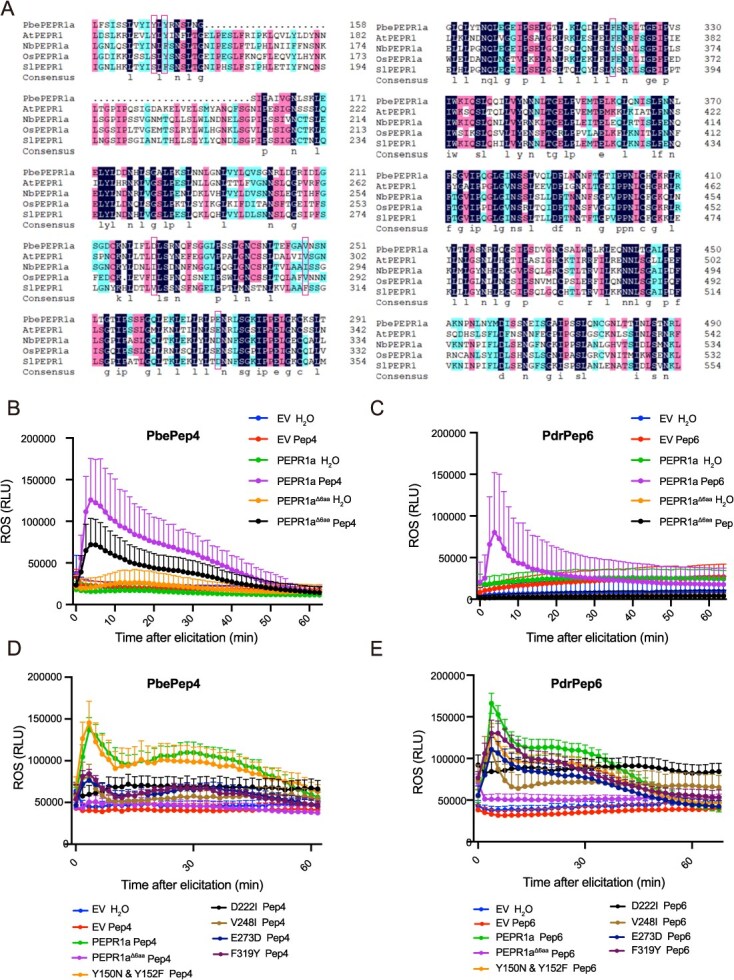
Key binding sites of PEPRs to the Peps contributed to the interfamily immunity. (A) Prediction of potential binding sites of PbePEPR1a-LRR to PbePep4 and PdrPep6 (red box). (B, C) ROS burst detected in tobacco leaf discs treated with PbePep4 (B) or PdrPep6 (C) after transient expression of PbePEPR1a and its mutated variant. The dynamic of ROS production of ROS accumulation were shown as mean values over 60 minutes ± SE. (D, E) ROS burst detected in tobacco leaf discs treated with PbePep4 (D) or PdrPep6 (E) after transient expression of PbePEPR1a and its mutated variants. The dynamic of ROS production of ROS accumulation were shown as mean values over 60 minutes ± SE.

In parallel, we compared the differences in the PEPR-LRR conformed domain between the PEPRs from Cruciferae and Rosaceae plants and those from Solanaceae plants and/or the differences in PbePEPR1a binding sites to PbePep4 and PdrPep6. The sequence alignment analysis indeed showed that there were six distinct sites of amino acids, including Y150, Y152, D222, V248, E273, and F319 of PbePEPR1a (Fig. 7A). Multiple mutation of these six residues of PEPR1a significantly reduced abolished ROS burst resulted from for the perception of PbePep4 (Fig. 7B) and PdrPep6 (Fig. 7C), respectively. Concerning the contribution of each site, we generated PbePEPR1a variants containing single mutation of these six residues. ROS measurement results demonstrated that among them, D222 plays the most important role in ROS burst (Fig. 7D and E), while other single mutations affected to a less extent in a Pep-dependent manner the perceptional function of PbePEPR1a for PbePep4 and PdrPep6. For PbePep4-induced ROS burst, D222 is pivotal, V248, E273, and F319 contributed less, while Y150 and Y152 had almost no role (Fig. 7D, Fig. S7), while for PdrPep6-induced ROS burst, all 6 amino acids seemed to play a role (Fig. 7E). These results suggested that the abovementioned six sites of PbePEPR1a work together for sensing pear Peps in a Pep-dependent manner.

Given that PbePEPR1a, tomato PEPR (*Solanum lycopersicum*, SlPEPR), and rice PEPR (*O. sativa*, OsPEPR) differ in these binding sites (Fig. 7A), we also investigated whether PbePep4 and PdrPep6 stimulate ROS burst in the presence of SlPEPR or OsPEPR in transient expression system. We found that neither SlPEPR nor OsPEPR initiated ROS burst upon treatment of PbePep4 or PdrPep6 (Fig. S6). Based on these results, we speculated that the differences in the PEPR-LRR conformed domain, especially for the binding sites to Peps, confers the distinct perception capacity of the PEPRs from between Cruciferae/Rosaceae and Solanaceae/Poaceae plants.

## Discussion

Many functional peptides have been identified in Rosaceae family plants, such as Rapid Alkalinization Factors (RALFs, [27]), phytosulfokines (PSKs, [27]) and C-terminally encoded peptides (CEPs) [28–30]. They are involved in pear growth, development, and reproduction. For instance, PbrPSK2 promotes pear pollen tube growth via relatively lower accumulation of ROS [27], while PbrRALF2 inhibits it through higher amount of ROS [31]. It is well known that Pep and RALF peptides are capable to enhance plant immunity by activating various plant immune responses, including ROS burst, callose deposition, MAPK phosphorylation, and defense genes expression [32]. Although Pep peptides in the Rosaceae family plants have been reported, the immune activation by Pep peptides has not yet been clarified. In this study, we demonstrated that: (i) seven PROPEPs and nine PEPRs were identified from five common pear varieties; (ii) PEPRs were expressed in the root, stem, and leaf; (iii) two representative pear Pep peptides, PbePep4 and PdrPep6, enhanced interfamily innate immunity in various plants by regulating ROS burst, callose deposition, MAPK phosphorylation, and defense genes expression. Our study laid a foundation for the study on Pep-driven resistance to necrotrophic diseases, including pear fire blight.

Pep-PEPR pairs are present in multiple species and the sequences of Peps and PEPR-LRRs appear to evolve very dynamically, resulting in a considerable divergence of the Pep-PEPR system [25, 33]. However, Pep peptides among the Solanaceae, Cruciferae, and Gramineaceae families, as well as between the Solanaceae and leguminous families, cannot stimulate plant immunity across families [24, 25]. A very recent study showed that Solanaceae Pep SlSolP12 could stimulate interfamily immunity in rice, soybean, and *Arabidopsis* [9]. In line with this study, we firstly demonstrated that two pear Peps can interfamilially enhance immunity against diverse plant diseases. Therefore, it is conceivable that Pep peptides with interfamily compatibility stimulate the immunity in broad spectrum of plants belonging to several families, which would save cost of plant disease control.

Specifically, PbePep4 and PdrPep6 can stimulate ROS surges in cruciferous plants (*Arabidopsis*, oilseed rape, and cabbage), as well as cucurbitaceae plants (watermelon, pumpkin, and loofah). By determining the family-specific Pep motifs, we proposed that the high similarity of the conformed motif at the C-terminal of Pep peptides from the Rosaceae, Brassicaceae, and Cucurbiaceae families determines interfamily compatibility. In parallel, given expressing transiently *AtPEPR1* and *PbePEPR1a* in *N. benthamiana* leaves, we speculated that the absence of key binding residues of PEPR-LRR conformed domain of the PEPRs from Cruciferae and Rosaceae leads to interfamily incompatibility [6, 24]. Therefore, it is possible to achieve cross-species recognition of Peps by replacing the key binding residues or certain LRR domains in PEPR1. Our study cannot rule out the possibility that other elements, such as co-receptors, may exist to determine the interfamily compatibility of PEP perception, which requires further investigation. Besides, whether Cucurbitaceae Peps can induce immunity in Rosaceae and cruciferous plants is an interesting topic to study in the future.

We also showed that application with PbePep4 and PdrPep6 interfamilially initiated typical responses of pattern triggered immunity (PTI, such as ROS burst, callose deposition, and MAPK phosphorylation) of the plants from Rosaceae, Brassicaceae, and Cucurbiaceae families. These findings supported that signaling machinery downstream of the PEPRs is highly conserved [24, 25, 34]). Our finding that PbePep4 and PdrPep6 interfamilially activate immune response and induce resistance to diverse pathogens in broad range of plant species significantly broadens their exploitation as immune activators and genes for molecular breeding for improved resistance.

Currently, emerging studies attempted to exploit Peps to improve plant innate immunity [23] or regeneration [8]. For instance, exogenous application of rice Pep OsPep3 improved the resistance to brown planthopper *Nilaparvata lugens*, strengthened rice resistance to the fungal pathogen *Magnaporthe oryzae* and bacterial pathogen *Xanthamonas oryzae* pv. *oryzae*, and provoked immune responses in wheat [7]. Treatment with SolP12 activates interfamily immunity in *Arabidopsis*, soybean, and rice [9]. Mixing Pep Systemin with the biocontrol fungus *Trichoderma afroharzianum* can enhance the resistance of tomato plants to *Fusarium spinae*, Botrytis and some pests [35]. Given that plant defense interconnected with regeneration facing wounding responses, various Peps (also called REF1) from different species boosts regeneration and transformation efficiency of corresponding crops [8]. Similarly, application of pear peptides PbePep4 and PdrPep6 can also be carried on in these directions, which will save a huge costs of plant disease prevention and control [5]. This study also provides evidence to a strategy that broadens the resistance utility of known peptides and enables breeders to redeploy these pear peptides across different plant families. Thus, the optimal inductance concentration of pear Pep peptides and the number of treatments requires further study. Attempts of generating transgenic plants harboring PbePep4 and PdrPep6 may also be beneficial to exploit the two broad-spectrum immune inducers.

## Materials and methods

### Plant materials and growth conditions

The seeds of pear Duli were provided by Dr Danying Cai from the Horticulture Research Institute of Zhejiang Academy of Agricultural Sciences. The seeds of watermelon 8424 were generously shared by Professor Fengming Song from Zhejiang University.

All *Arabidopsis thaliana* plants used in this study were in Columbia-0 background. The seeds were sterilized with 15% sodium hypochlorite for 5 minutes and 75% (v/v) ethanol for 5 minutes, washed three times with sterile water, and grown on half-strength Murashige and Skoog (MS) agar plates in a growth chamber under a 16-hour photoperiod with 70% humidity at 22°C. Three-week-old seedlings were used for young seedling assays or transplanted to soil for further growth.

### Plasmid construction and plant transformation

To construct plasmids with point mutations of PbePEPR1a, primers with the desired mutations were used in a PCR reaction to amplify variants. The primers used to generate the constructs are listed in Table S3.

### Synthetic peptides and elicitors

The PbePep4 (DEAAAITRIKVSARERPGLSTGKGGKTN) and PdrPep6 (MAAARSATRVPTSIARARPKNHNKPPLSSGKGGQIN) peptides were synthesized by Qiangyao (China) with a purity of ≥95%. All peptides were dissolved in sterile water.

### Plant inoculation and fungal biomass assays

Pear fire blight: One micromolar of PbePep4 or PdrPep6 with the additive lauryl glucoside was sprayed on pear leaves, which were then inoculated 24 hours later. The strain of *E. amylovora* stored at −80°C were spread on NA solid medium and cultured at 28°C for 1 day. The monoclonal cells were picked and placed in 2 ml EP tubes containing 400 μl NB liquid medium and shaken at 28°C overnight. The ratio of the bacterial solution to the NA liquid medium was 10:1 for medium culture. When OD_600_ arrived 0.6 to 0.8, culture was centrifugated at 5,000 rpm for 6 minutes and then resuspended to OD_600_ 0.5. The needle was soaked in the bacterial liquid for 30 minutes. The veins of the pear leaves were pricked with the needle for inoculation. Pear leaves were kept in moist chamber at 27°C to observe the disease occurrence. The diseased leaves were disinfected twice with 75% ethanol and rinsed three times with sterile water. Circular pieces of the diseased leaves were punched with a 3 mm perforator and placed in a 2-ml EP tube. One hundred microliters of ddH_2_O was added into the EP tube. Leaves were ground for 2 minutes. Ten microliters of the liquid was aspirated for gradient dilution and other 10 μl was aspirated and placed on the NA solid medium. The plate was incubated at 28°C for 2 days to observe strain growth.

Oilseed rape Sclerotinia stem rot: Fresh sclerotia of *Sclerotinia sclerotiorum* were cultured at 23°C on potato dextrose agar (PDA) medium to produce mycelia, which were transferred to new PDA plates and grown for 2 days. The PDA plugs containing newly developed mycelia were punched to inoculate the plant leaves. Oilseed rape leaves were pretreated by spraying 1 μM of PbePep4 or PdrPep6 with the adjuvant lauryl glucoside for 24 hours. The leaves were cut and placed in inoculation box lined with wet paper towels for 24 hours.

Watermelon gray mold: *Botrytis cinnerea* was inoculated onto the V8 solid medium and cultured in an incubator at 25°C for 2 weeks. When the mycelium turned gray, the mycelium cake was obtained and placed in the spore suspension (4% maltose, 1% peptone), crushed and filtered out impurities with gauze. The collected spore suspension was diluted to a concentration of 1 × 10^5^ spores per ml. The hemocytometer has ~10 spores in each large square. Watermelon leaves were pretreated by spraying 1 μM of PbePep4 and PdrPep6 with lauryl glucoside for 24 hours. The leaves were cut and placed in inoculation box lined with wet paper towels. Two spots were inoculated on each leaf, with 2.5 μl of spore suspension at each spot. The leaves were covered with film for moisture retention and cultivated at 23°C for ~3 days. Lesion areas were measured using ImageJ software (https://imagej.net).

### ROS detection

ROS production was measured using a chemiluminescence assay as previously described with some modifications [36]. Leaf disks (3 mm in diameter) were sampled and incubated overnight in 96-well plates with sterile water. At least eight plants per genotype were examined. A mixture containing 11 mM L-012 (Wako Chemicals, Japan), 20 mg/ml horseradish peroxidase (Sigma Aldrich), and 1 μM PbePep4 or PdrPep6 or 100 nM flg22 peptide was replaced before chemiluminescence detection with a Microplate Luminometer (Titertek Berthold, Germany). Chemiluminescence signals were recorded for the indicated time period.

To observe H_2_O_2_  *in situ*, a DAB staining assay was performed as described previously with some modifications [37]. Leaves infiltrated with peptides were collected at 2 hours post-infiltration and soaked in DAB solution (1 mg/ml, pH 3.5) for 3 hours. Chlorophyll was removed using 95% ethanol, and the reddish-brown color indicative of H_2_O_2_ production was observed under a light microscope (Nikon, Japan). The relative intensity of staining was measured using ImageJ software.

### Cytosolic calcium measurements

The procedures for aequorin-based [Ca^2+^]_cyt_ measurements followed the published method [38] with some modifications. Seven-day-old aequorin transgenic seedlings grown vertically on half-strength MS medium were transferred to 96-well plates containing 50 ml of 10 mM coelenterazine h (Sigma) solution and incubated overnight in the dark at room temperature. An additional 50 ml of ddH_2_O containing 1 μM PbePep4 or PdrPep6 peptides was supplied before measurement. The dynamics of photon counts, which were converted to [Ca^2+^]_cyt_, were recorded immediately using a Microplate Luminometer (Titertek Berthold, Germany).

### Callose deposition assay

Callose deposition was measured as previously described with some modifications [36]. Three-week-old leaves of mature plants infiltrated with 1 μM PbePep4 or PdrPep6 were collected at 24 hours post-infiltration and washed twice with ddH_2_O and ethanol. After removal of chlorophyll with acetic acid and ethanol (1:3) for 4 hours, leaves were washed twice with ddH_2_O and stained with aniline blue solution (150 mM KH_2_PO_4_, 0.1% [w/v] aniline blue, pH 9.5). Callose deposition was observed under UV excitation, and images were photographed using a stereomicroscope (Nikon). The intensity was measured using ImageJ software.

### DAB staining

One micromolar pear PbePep4 or PdrPep6 peptide was used to soak the leaves. After 24 hours, the leaves were cut off. At room temperature, the leaves were stained in the dark for 4 hours in a DAB solution 1 mg/ml (pH 3.8). They were decolorized with 95% (v/v) ethanol. After complete decolorization of the leaves, photos were taken for recording and statistical analysis.

### Mitogen-activated protein kinase phosphorylation analysis

Mitogen-activated protein kinase (MAPK) phosphorylation analysis was performed as previously described with some modifications [36, 38]. Total protein was extracted using a buffer (50 mmol/l pH 7.5 Tris–HCl, 150 mmol/l NaCl, and 1% Triton X-100) containing protease inhibitors and the phosphatase inhibitor calyculin. Proteins were separated by 12.5% SDS-PAGE and transferred to polyvinylidene difluoride membranes (General Electric Company, USA) at 90 V for 1.5 hours at room temperature. After blocking with 4% bovine serum albumin (BSA), membranes were incubated with an antiphospho-p44/p42 MAP kinase antibody (Cell Signaling Technology, USA). Signals were detected using Novex® Chemiluminescent Substrates (ThermoFisher, USA), and Ponceau S staining was used for the loading control.

### Agrobactrium-mediated gene expression assay

The transient gene expression in tobacco was activated by preserving the GV3101 bacterial solution. A small amount of single-colony culture was carried out. The bacteria were shaken overnight at 28°C and 220 rpm. Four-milliliter culture was shaken at a ratio of 1:10 and at 28°C and 220 rpm for 4 hours until OD_600_ reaching 0.8 to 0.9. The culture was centrifugated at 5,000 rpm at room temperature for 8 minutes and the resuspended with MMAi to control OD_600_ at 0.5 to 1.0. After recovering at 28°C for 1.5 hours, the bacterial liquid was injected into the back of tobacco leaves with a 1-ml syringe. Subsequent experiments such as Pep infiltration were conducted after 48 hours.

### RNA extraction and qRT–PCR analysis

Total RNA was extracted from leaves with or without peptide treatments and *S. sclerotiorum* inoculation at the indicated time points using TRIzol (Vazyme, China) according to the manufacturer’s instructions. The procedures and kits used for qRT–PCR were described previously [36]. *AtActin8* and *BnActin7* were used as internal control genes in *A. thaliana* and *B. napus*, respectively. Relative gene expression was calculated using the 2^−△△^Ct method as recommended by the manufacturer. All primers used for qRT-PCR are listed in Table S3. Quantification was performed with three biological replicates per sample.

### Analysis of antibiotic effects of Pep peptides

*Erwinia amylovora*: Single colony of *E. amylovora* strain were picked and cultured in small quantities in NB liquid medium. Thirty milliliters of the bacterial culture was incubated at a ratio of 10:1 of the bacterial solution and NA liquid medium. The culture continued until the OD_600_ was 0.6 to 0.8, and the bacterial solution was diluted to an OD_600_ of 0.2. One micromolar of pear PbePep4 or PdrPep6 was added into the culture. Same volume of ddH_2_O was used as the negative control. The dynamic process of fine growth was monitored using the fully automatic cell imaging multifunctional microplate detector (BioTek Cytation 1, Germany) at a temperature of 28°C. The OD600 value of the bacterial solution was recorded once every 1 hour. Afterwards, bacterial solution was diluted by two times and then by 10 times. Five microliters of the bacterial solution was placed on the NA solid medium. Plate was placed at 28°C for 2 days. The growth of the colonies was recorded.

*Sclerotinia sclerotiorum*: The activated *S. sclerotiorum* was picked as 3 -mm discs and placed at the center of 90-mm circular PDA solid medium. The plate was placed at 25°C for 18 hours. Four 3-mm holes were made along the edge, and 1 μM of pear PbePep4 or PdrPep6 were added into. ddH_2_O was used as the negative control. The plate was incubated at 25°C until the mycelium grew to the edge of the PDA solid medium.

*Botrytis cinerea*: One micromolar of pear PbePep4 or PdrPep6 was added into the V8 medium. The activated *B. cinerea* discs were added into the V8 medium and incubated at 25°C for 2 weeks to prepare a spore suspension. The growth and development of the spores were observed under a microscope.

### Phylogenetic tree construction

The database used in this study are Rosaceae genome database (https://www.rosaceae.org/) and Cucurbitaceae genome database (http://cucurbitgenomics.org/). The phylogenetic tree of PROPEP and PEPR protein sequences of the Rosaceae family was constructed using MEGA 12 software. The homology comparison of proteins was performed using the CLC Main workbench software.

### Molecular docking

The amino acid sequences of PbePEP4 and PEPRs were used as input for protein complex prediction. Protein complexes were modeled using AlphaFold 3.0 [39] using default settings. All the figures representing structures were prepared with PyMOL.

### Statistical analysis

Statistical significance was determined using Student's *t*-test or one-way ANOVA and GraphPad Prism 8.0 software.

## Consent for publication

All authors approve the manuscript and consent to the publication of the work.

## Supplementary Material

Web_Material_uhag027

## Data Availability

All pertinent data are included within the manuscript and accompanying supplementary information files.
