# Supplementary material for: Two pear phytocytokines PbePep4 and PdrPep6 interfamilially elicit broad-spectrum immunity against various pathogens in crops
Source: Hortic Res. 2026 Jan 29;13(5):uhag027. doi: 10.1093/hr/uhag027 (PMC13148170; doi:10.1093/hr/uhag027)
Supplement: Web_Material_uhag027 [file web_material_uhag027.zip › Revised-Supplementary Figures for HR.docx]

**Supplementary Figures**


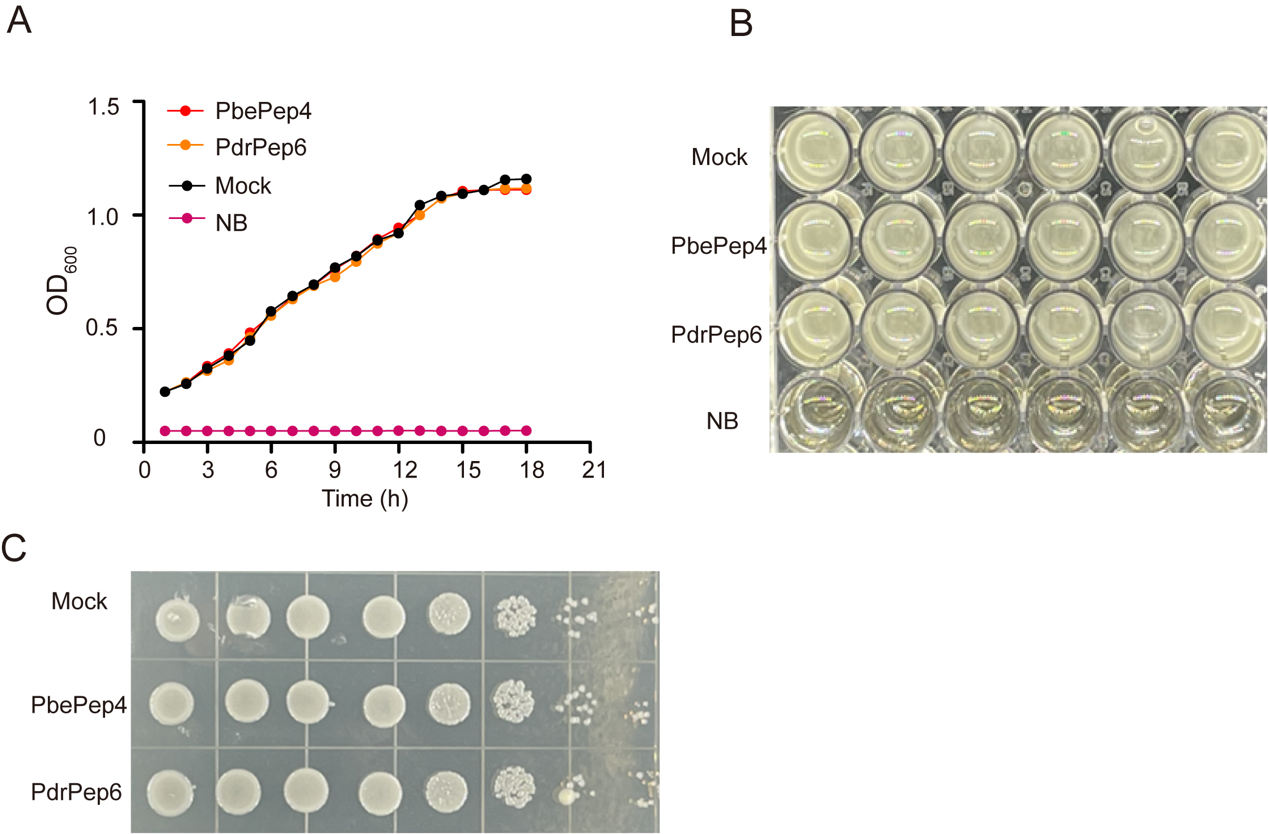


Figure S1 PbePep4 and PdrPep6 did not have antibiotic effect on *E. amylovora*

**A**. Dynamic of *E. amylovora* growth after treated with PbePep4 and PdrPep6. The initial OD_600_ of bacterial solution was approximately 0.2 and NB was used as a negative control.

**B**. *E. amylovora* solution was cultured for 18 h.

**C**. Growth of the bacterial solution in **B** after a 10-fold gradient dilution on NA solid medium for 36 h.

For **A**, **B**, and **C**, the experiments were repeated three times.


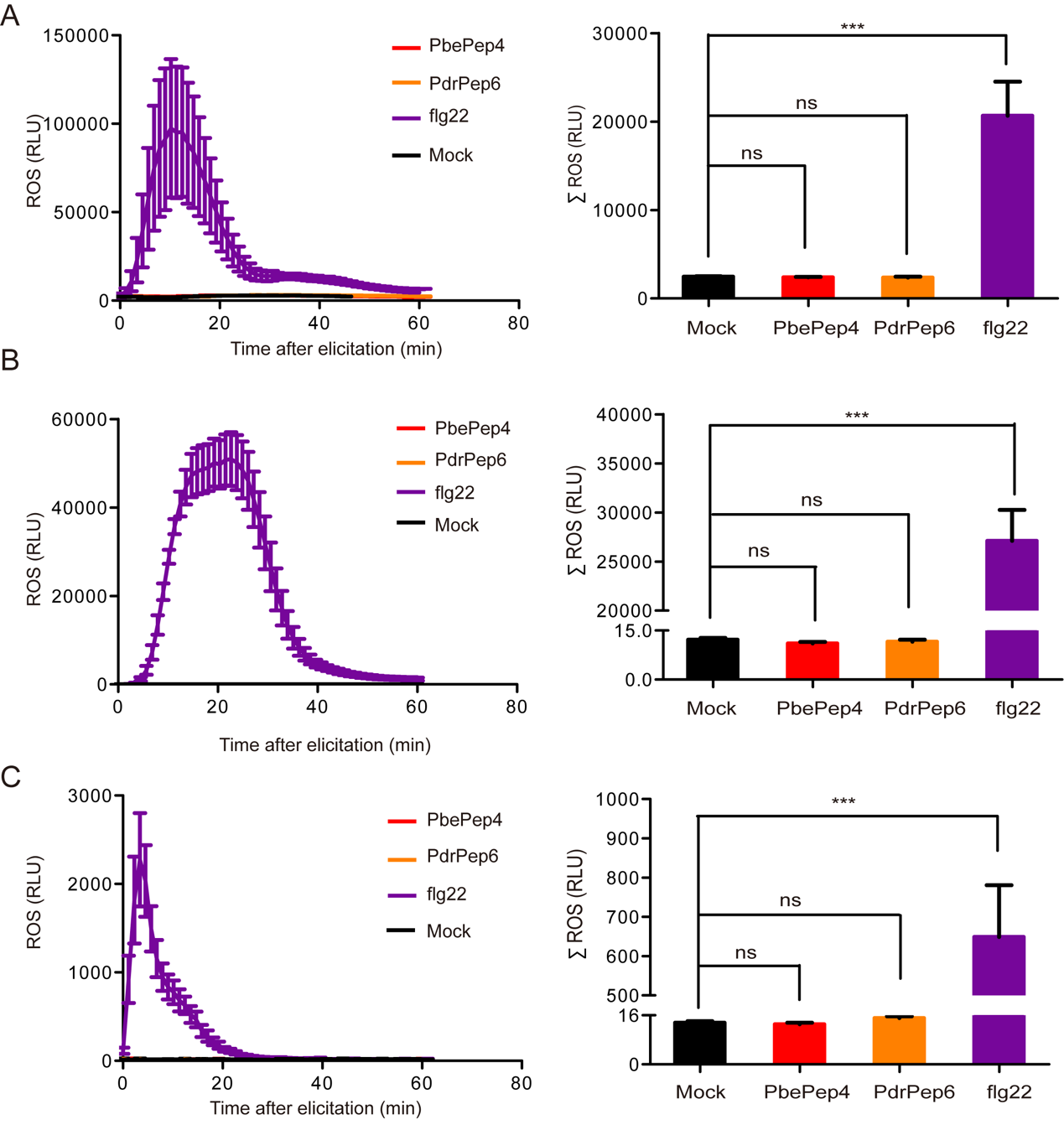


Figure S2 PbePep4 and PdrPep6 did not elicit ROS burst in Solanaceae species

**A**, **B**, and **C**. ROS burst detected in *S. lycopersicum* (**A**), *N. tabacum* (**B**) and *C. annuum* (**C**) leaf discs respectively treated with PbePep4 and PdrPep6. The dynamic of ROS production (left panel) and statistics of ROS accumulation (right panel) were shown as mean values over 60 min ± SE. ddH_2_O was used as a negative control and flg22 as a positive control.

The experiments were repeated at least three times. One-way ANOVA method was used to test the significance of differences among experimental groups. For **A**, **B**, and **C**, the data were shown as the mean ± SE. Star numbers represent levels of difference significance (***, *P* < 0.001, ns, not significant).


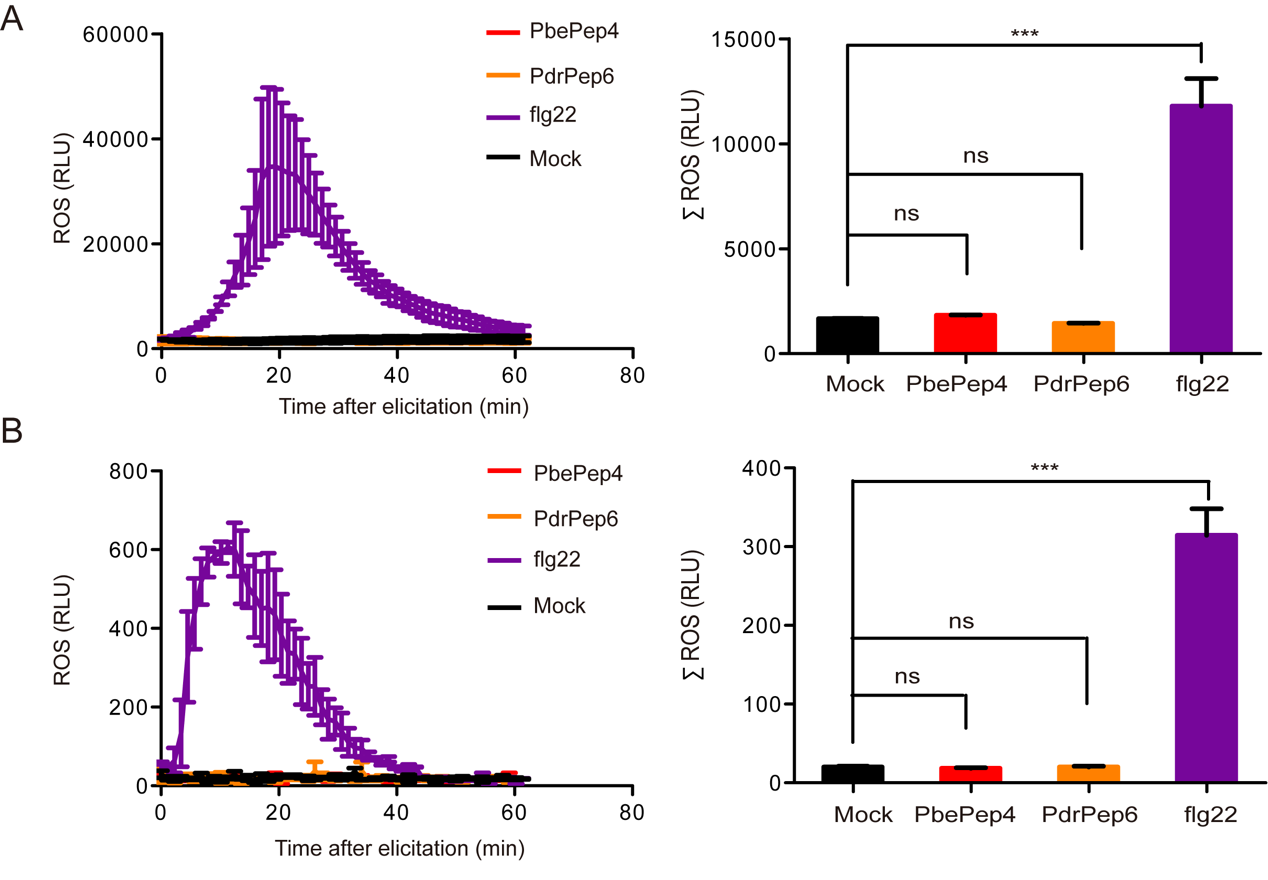


Figure S3 PbePep4 and PdrPep6 did not elicit ROS burst in Poaceae species

**A**, **B**. ROS burst detected in *O. sativa* (**A**) and *Z. mays* (**B**) leaf discs respectively treated with PbePep4 and PdrPep6. The dynamic of ROS production (left panel) and statistics of ROS accumulation (right panel) were shown as mean values over 60 min ± SE. ddH_2_O was used as a negative control and flg22 as a positive control.

The experiments were repeated at least three times. One-way ANOVA method was used to test the significance of differences among experimental groups. For **A** and **B**, the data were shown as the mean ± SE. Star numbers represented levels of difference significance (***, *P* < 0.001, ns, not significant).


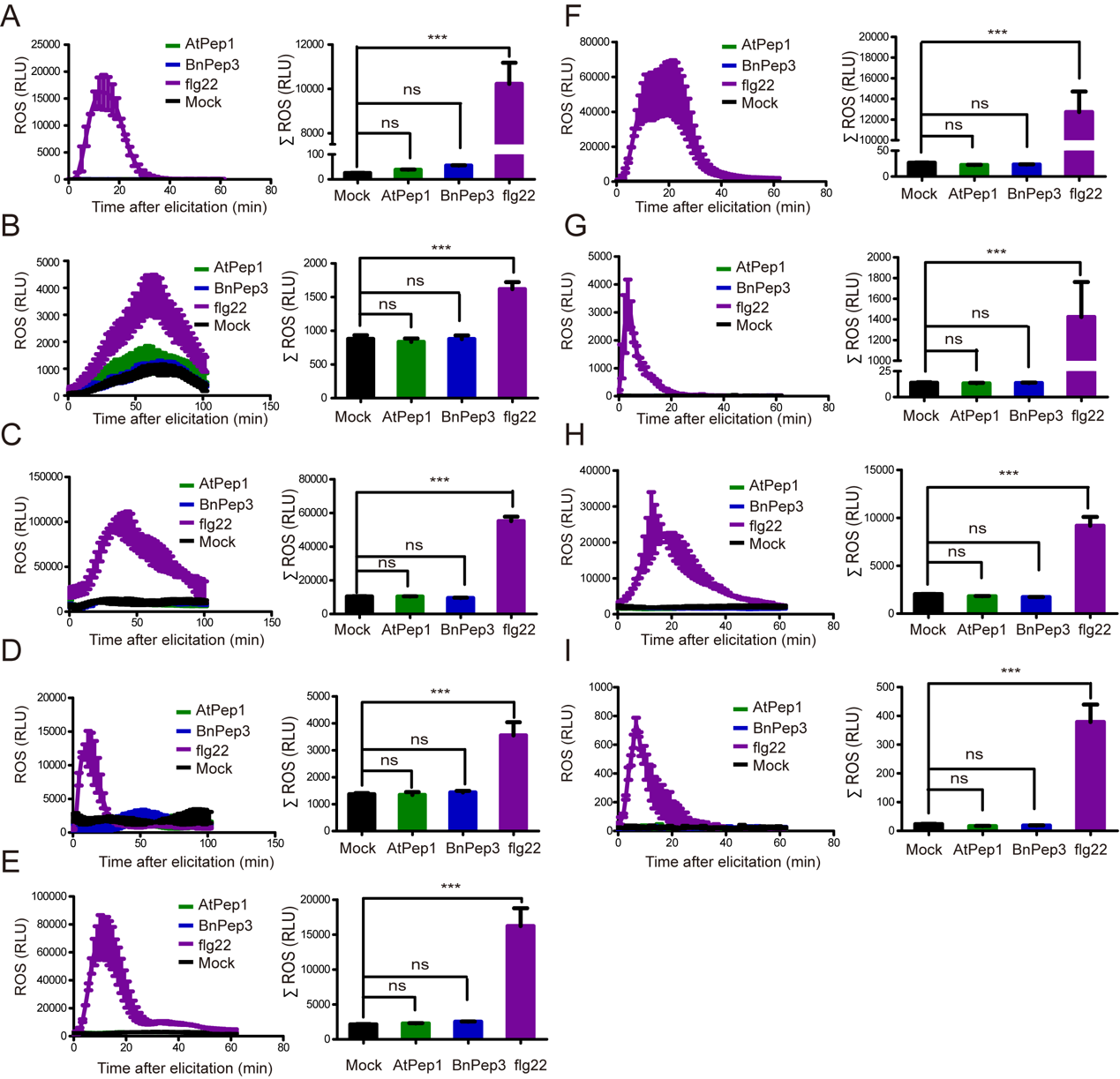


Figure S4 AtPep1 and BnPep3 did not elicit ROS burst in Rosaceae, Cucurbitaceae, Solanaceae, and Poaceae species

**A**-**I**. ROS burst detected in the leaf discs of *P. betulifolia* (**A**), *C. lanatus* (**B**), *C. moschata* (**C**), *L. aegyptiaca* (**D**), *S. lycopersicum* (**E**), *N. tabacum* (**F**), *C. annuum* (**G**), *O. sativa* (**H**), and *Z. mays* (**I**) treated with AtPep1 and BnPep3. The dynamic of ROS production (left panel) and statistics of ROS accumulation (right panel) were shown as mean values over 60 min ± SE. ddH_2_O was used as a negative control and flg22 as a positive control.

The experiments were repeated at least three times. One-way ANOVA method was used to test the significance of differences among experimental groups. For **A-I**, the data were shown as the mean ± SE. Star numbers represented levels of difference significance (***, *P* < 0.001, ns, not significant).


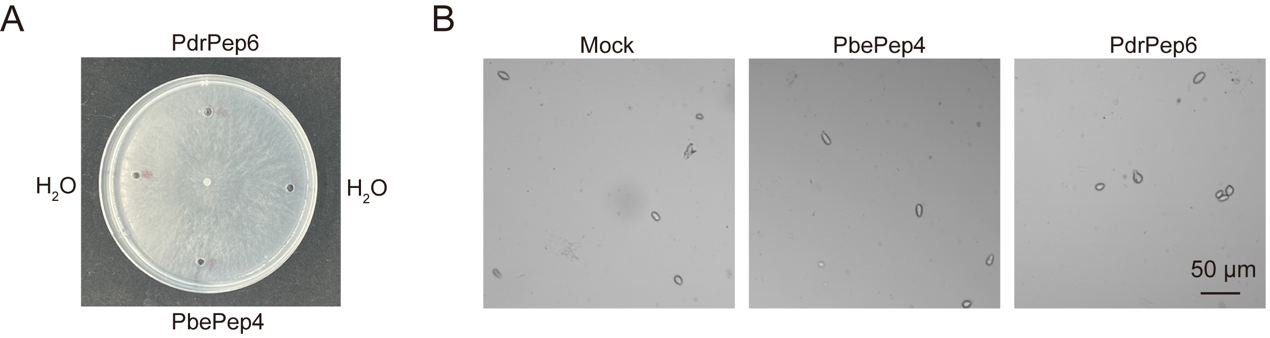


Figure S5 PbePep4 and PdrPep6 did not have antibacterial effect on *S. sclerotiorum* and *B. cinerea*

A. Effect of PbePep4 and PdrPep6 treatment on the growth of *S. sclerotiorum*. *S. sclerotiorum* was inoculated on PDA medium. Three-mm in diameter holes were drilled in the plate, which were added with PbePep4 and PdrPep6. The mycelia growth was observed.

**B.** Effect of PbePep4 and PdrPep6 treatment on the growth of *B. cinerea*. *B. cinerea* was inoculated on V8 solid medium containing PbePep4 and PdrPep6 and cultured for two weeks to prepare spore suspension. The growth of conidium was observed under microscope.

For **A** and **B**, the experiments were repeated three times.


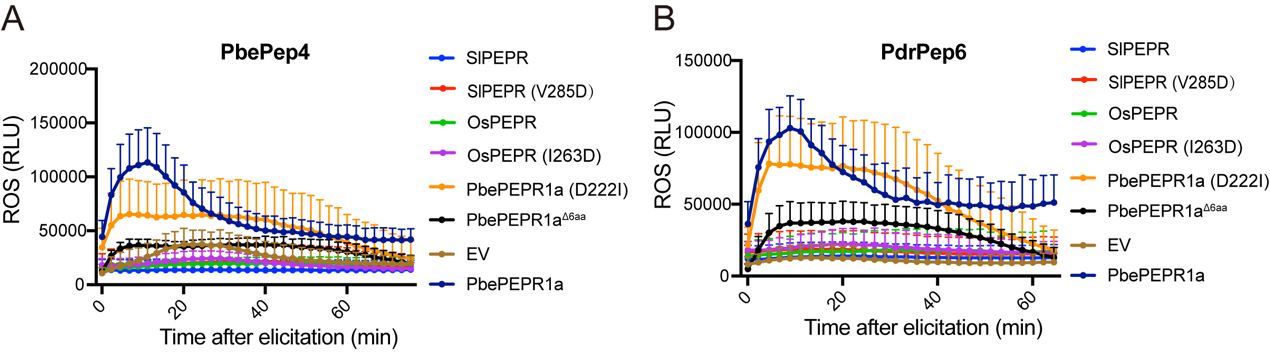


Figure S6 SlPEPR and OsPEPR could not sense pear peptides PbePep4 and PdrPep6

**A**, **B**. ROS burst detected in tobacco leaf discs treated with PbePep4 (**A**) or PdrPep6 (**B**) after transient expression of PbePEPR1a, SlPEPR, and, OsPEPR and their mutated variant. The dynamic of ROS production of ROS accumulation were shown as mean values over 60 min ± SE.


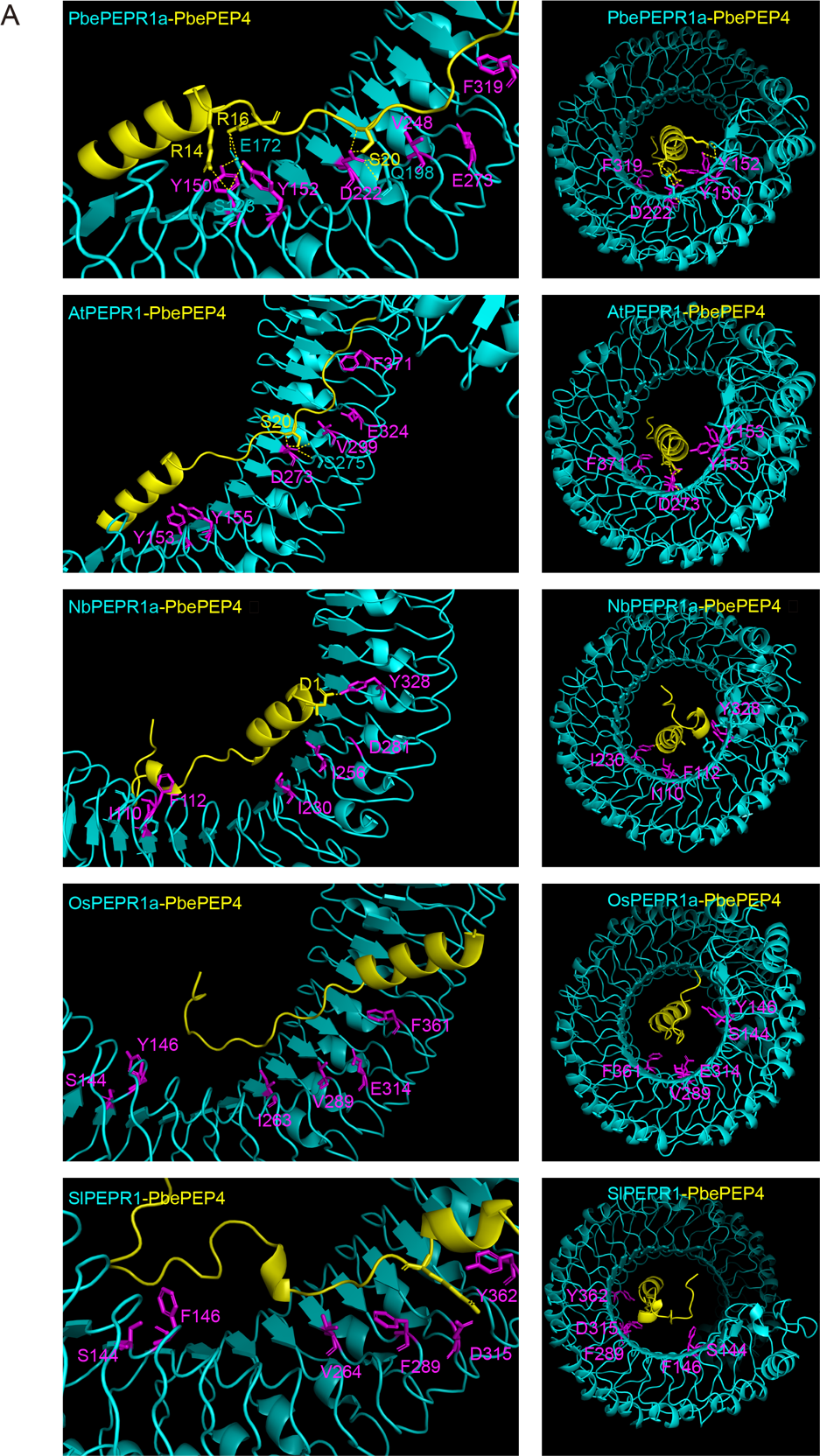


Figure S7. Molecular docking of PbePep4 with PbePEPR1a, AtPEPR1, NbPEPR1a, OsPEPR1a, and SlPEPR1.

**A**, Close-up views of the interaction sites in structural models of the complexes of PbePep4 with PEPRs, as predicted by AlphaFold 3.0.
