# Supplementary material for: Two pear phytocytokines PbePep4 and PdrPep6 interfamilially elicit broad-spectrum immunity against various pathogens in crops
Source: Hortic Res. 2026 Jan 29;13(5):uhag027. doi: 10.1093/hr/uhag027 (PMC13148170; doi:10.1093/hr/uhag027)
Supplement: Web_Material_uhag027 [file web_material_uhag027.zip › Revised-Supplementary Tables for HR.docx]

**Supplementary Tables**

Table S1 Pep peptides of *Pyrus*

| Species | Name | Full precursor | Nature Pep | Length | ID |
| --- | --- | --- | --- | --- | --- |
| *Pyrus betulifolia* | PbePep4 | MEYSLSHKGSADEVEELMDKRSYYIFPCHFLEEAVRAFFKCLGIETKSQEDKDTDEINPEKITPPTSEQLLDGAVPSLLMTYGDPPSSSATETADEAAAITRIKVSARERPGLSTGKGGKTN | DEAAAITRIKVSARERPGLSTGKGGKTN | 28 | GWHTAAYT040784 |
| *Pyrus pyrifolia* Cuiguan | PpcPep4 | MEYSQSSKVLADDEEELMEKRSYRIFPCHFLEEAVRAFFKCLGIETKYQEDKENPKEATPASTPEQNDADPSLPTTEDDPPSSSATTGTTDEVAANTTTNLRARARPGLSTGGGGRINSLNN | DEAAAITRIKVSARERPGLSTGKGGKTN | 28 | GWHTBAOS002281 |
| P*yrus pyrifolia* Cuiguan | PpcPep6 | MSSQAEEEEEEEEERQGEQARHRFYYSPCSLFEHAIKAFLKCLGLHDDHHDYHSGGSAVTDDQNHHHHPHPHPHPHRDPHTEREMKFTEEAVIVMAAARSATRVPTSIARARPKNHNKPPLSSGKGGQIN | MAAARSATRVPTSIARARPKNHNKPPLSSGKGGQIN | 36 | GWHTBAOS014519 |
| *Pyrus pyrifolia* | PpyPep4 | MEYSQSSKVLADDEEELMEKRSYRIFPCHFLEEAVRAFFKCLGIETKYQEDKENPKEATPASTPEQNDADPSLPTTEDDPPSSSATTGTTDEVAANTTTNLRARARPGLSTGGGGRINSLNN | DEVAANTTNLRARARPGLSTGGRIN | 25 | Ppy00g1015.1 |
| *Pyrus ussuriensis* x *communis* Zhongai | PdrPep4 | MEYSQSSKVLADDEEELMEKRSYRIFPCHFLEEAVRAFFKCLGIETKYQEDKENPKEATPASTPEQNDADPSLPTTEDDPPSSSATTGTTDEVAANTTTNLRARARPGLSTGGGGRINSLNN | DEVAANTTTNLRARARPGLSTGGGGRIN | 28 | Pdr10g026060.1 |
| *Pyrus ussuriensis* x *communis* Zhongai | PdrPep6 | MSSQAEEEEERQGEQARHRFYYSPCSLFEHAIKAFLKCLGLHDDHHDYHSGGSAFTDDQNHHHHPHPHRDPHTEREMKFTEEAVIVMAAARSATRVPTSIARARPKNHNKPPLSSGKGGQIN | MAAARSATRVPTSIARARPKNHNKPPLSSGKGGQIN | 36 | Pdr15g005090.1 |
| *Pyrus communis* | PycPep4 | MEYSLSHKVSADEEEELMDKRSYYIFPCHFLEEAVRAFFKCLGIETKSQEDKDTDEINPEKITPPTSEQLLDGAVPSLLMTYGDPPSSSATETADEAAAITRIKVSARERPGLSTGKGGKTN | DEAAAITRIKVSARERPGLSTGKGGKTN | 28 | pycom05g03040 |

Table S2 Identification result of PEPR from *Pyrus*

| **Species** | **Name** | **ID** |
| --- | --- | --- |
| *Pyrus betulifolia* | PbePEPR1a | GWHPAAYT029331 |
| *Pyrus betulifolia* | PbePEPR1b | GWHPAAYT0S4814 |
| *Pyrus communis* | PycPEPR1a | pycoml7g13740 |
| *Pyrus communis* | PycPEPR1b | pycom09g07690 |
| *Pyrus pyrifolia* Cuiguan | PpcPEPR1a | GWHPBAOS020897 |
| *Pyrus pyrifolia* | PpyPEPR1a | Ppy17g1334.1 |
| *Pyrus pyrifolia* | PpyPEPR1b | Ppy09g1276.1 |
| *Pyrus ussuriensis* x *communis* Zhongai | PdrPEPR1a | Pdr17g008810.1 |
| *Pyrus ussuriensis* x *communis* Zhongai | PdrPEPR1b | Pdr9g010510.1 |

**Table S3 Primers used in this study**

| **Use** | **Name** | **Sequence** |
| --- | --- | --- |
| pCambia-1305-GFP  constructs | GFP-PbePEPR1a-150Y-F | TCGATTTCCAGCTTGGTTAATATATTTCTGTATAGGA |
|  | GFP-PbePEPR1a-150Y-R | TCCTATACAGAAATATATTAACCAAGCTGGAAATCGA |
|  | GFP-PbePEPR1a-222D-F | ATTTGATTTTCTTGATTTTGTCACGCAAT |
|  | GFP-PbePEPR1a-222D-R | ATTGCGTGACAAAATCAAGAAAATCAAAT |
|  | GFP-PbePEPR1a-248V-F | GTTTGGTGCTATCAATAGCAACTT |
|  | GFP-PbePEPR1a-248V-R | AAGTTGCTATTGATAGCACCAAAC |
|  | GFP-PbePEPR1a-273E-F | TGCGCCTTCCTGATAACCGTTTGTCT |
|  | GFP-PbePEPR1a-273E-R | AGACAAACGGTTATCAGGAAGGCGCA |
|  | GFP-PbePEPR1a-319F-F | ATCTTGAATTGTATGAGAACAGGTTA |
|  | GFP-PbePEPR1a-319F-R | TAACCTGTTCTCATACAATTCAAGAT |
|  | 1305-GFP-PbePEPR1a-F | aagtccggagctagctctagaATGAAGCTTTACCCTTTCAATTTCTT |
|  | 1305-GFP-PbePEPR1a-R | gcccttgctcaccatggatccGCCCTTTATGCTTCTCACTTTGG |
|  | NbPEPR-GFP-F | aagtccggagctagctctagaATGAATACTGCTTTTAGAAATTTCATC |
|  | NbPEPR-GFP-R | gcccttgctcaccatggatccTGATGTTGAAAGCGTCTGCG |
|  | NbPEPR-265F | TGACAATCTGGACTTGTCAGACAAC |
|  | NbPEPR-265R | GTTGTCTGACAAGTCCAGATTGTCA |
|  | OsPEPR-GFP-F | aagtccggagctagctctagaATGAGGCTGGTTGTGTGGCA |
|  | OsPEPR-GFP-R | gcccttgctcaccatggatccGGACGAGCTCCGGGAGGC |
|  | OsPEPR-263F | TGAGGTATTCGACTTGTCATTCAATC |
|  | OsPEPR-263R | GATTGAATGACAAGTCGAATACCTCA |
|  | SlPEPR-GFP-F | aagtccggagctagctctagaATGCTACCTAAACTCATATCTTTTTCT |
|  | SlPEPR-GFP-R | gcccttgctcaccatggatccGTACTTGCTTCGTATACTCGAACTTGA |
|  | SlPEPR-285F | TCGACACTCTGGACTTGTCATCCAAT |
|  | SlPEPR-285R | ATTGGATGACAAGTCCAGAGTGTCGA |
| RT-qPCR | PbrUBQ-qRT-F | GCACAAGAAGGTGAAGCTCG |
|  | PbrUBQ-qRT-R | ACTCAGCATTGGGGCACTC |
|  | Pbr9127-qRT-F | GAACGCTCCTTTCTGCATGTTT |
|  | Pbr9127-qRT-R | TCGTTTTCTTTCCCGCAATCAC |
|  | Pbr9943-qRT-F | CCCGAGAACGCCAAATGTGGAG |
|  | Pbr9943-qRT-R | GGACCTCCTCCACCTCTAAGTAA |
| Pathogen quantification | Rubisco-qPCR-F | GCAAGTGTTGGGTTCAAAGCTGGTG |
|  | Rubisco-qPCR-R | CCAGGTTGAGGAGTTACTCGGAATGCTG |
|  | Sc ITS -qPCR-F | GGATCTCTTGGTTCTGGCAT |
|  | Sc ITS-qPCR-R | GCAATGTGCGTTCAAAGATT |
